# Pitting of malaria parasites in microfluidic devices mimicking spleen interendothelial slits

**DOI:** 10.1038/s41598-021-01568-w

**Published:** 2021-11-11

**Authors:** Aleix Elizalde-Torrent, Claudia Trejo-Soto, Lourdes Méndez-Mora, Marc Nicolau, Oihane Ezama, Melisa Gualdrón-López, Carmen Fernández-Becerra, Tomás Alarcón, Aurora Hernández-Machado, Hernando A. del Portillo

**Affiliations:** 1 ISGlobal Institute for Global Health, Hospital Clínic - Universitat de Barcelona, Barcelona, Spain; 2 Instituto de Física, Pontificia Universidad Católica de Valparaíso, 4059 Casilla, Chile; 3 Department of Condensed Matter Physics, University of Barcelona (UB), Barcelona, Spain; 4 IGTP Institut d’Investigació Germans Trias I Pujol, Badalona, Barcelona Spain; 5 ICREA: Catalan Institution for Research and Advanced Studies, Barcelona, Spain; 6 Centre de Recerca Matemàtica (CRM), Bellaterra, Barcelona Spain; 7 Departament de Matemàtiques, Universitat Autònoma de Barcelona, Bellaterra, Barcelona Spain; 8 Institute of Nanoscience and Nanotechnology (IN2UB), University of Barcelona (UB), Barcelona, Spain; 9 Present Address: IrsiCaixa AIDS Research Institute, Badalona, Spain

**Keywords:** Malaria, Biophysics, Medical research, Biomedical engineering, Biological physics

## Abstract

The spleen is a hematopoietic organ that participates in cellular and humoral immunity. It also serves as a quality control mechanism for removing senescent and/or poorly deformable red blood cells (RBCs) from circulation. Pitting is a specialized process by which the spleen extracts particles, including malaria parasites, from within circulating RBCs during their passage through the interendothelial slits (IES) in the splenic cords. To study this physiological function in vitro, we have developed two microfluidic devices modeling the IES, according to the hypothesis that at a certain range of mechanical stress on the RBC, regulated through both slit size and blood flow, would force it undergo the pitting process without affecting the cell integrity. To prove its functionality in replicating pitting of malaria parasites, we have performed a characterization of *P. falciparum*-infected RBCs (*P.f.*-RBCs) after their passage through the devices, determining hemolysis and the proportion of once-infected RBCs (O-iRBCs), defined by the presence of a parasite antigen and absence of DAPI staining of parasite DNA using a flow cytometry-based approach. The passage of *P.f.*-RBCs through the devices at the physiological flow rate did not affect cell integrity and resulted in an increase of the frequency of O-iRBCs. Both microfluidic device models were capable to replicate the pitting of *P.f.*-RBCs ex vivo by means of mechanical constraints without cellular involvement, shedding new insights on the role of the spleen in the pathophysiology of malaria.

## Introduction

The spleen is a secondary lymphoid organ that performs critical physiological functions ranging from induction of adaptive cellular and humoral immune response, iron recycling, removal from circulation of senescent red blood cells (RBCs), bacteria, and other pathogens, including malaria parasites^1^. The capacity to fulfill its blood filtering function is given to the exquisite tissue compartmentalization and its unique circulatory system.

The human spleen is organized in two functional compartments known as white pulp and red pulp separated by a marginal zone. The white pulp is composed by T cells lining around arterioles surrounded by a B cells zone forming follicles. The red pulp represents the large majority of the spleen tissue and is formed by reticular cells, and immune cells which function in monitoring aged, dead or opsonized RBCs and in the surveillance of pathogens^1^. Importantly, the red pulp contains circulation open spaces lacking endothelial lining called splenic cords, which are populated by reticular fibers and reticular cells forming a complex mesh. Blood is delivered from terminal arterioles into the splenic cords, where highly active macrophages survey circulating cells and particles to remove aged, infected or dysfunctional RBCs. Before exiting this compartment, in sinusoidal spleens such as that of humans, RBCs pass through interendothelial slits (IES), 1–2 micron structures of the venous sinuses, before reentering into circulation.

During this stringent quality control process of senescent and/or poorly deformable RBCs, the spleen also removes particles from within circulating red cells such as nuclear remnants (Howell-Jolly bodies), insoluble globin precipitates (Heinz bodies), normally-occurring endocytic vacuoles^2^ and malaria parasites^3^. This phenomenon, known as pitting, describes the squeeze passage of erythrocytes containing inclusion bodies through the narrow IES of the venous sinus wall, removing them and leaving a fraction of the cell containing the non-deformable inclusion bodies trailing behind the reticular meshwork side. Therefore, pitting refers to a corrective mechanism rather than the removal of defective RBCs through phagocytosis which is sometimes referred as the culling function of the spleen^2^.

For ethical and technical reasons, functional studies of the spleen have been limited to animal models^1^. The development and validation of lab-on-a-chip microfluidic devices to accelerate physiological and drug discovery and to reduce animal experimentation^4,5^, offers a robust alternative to study the function of the spleen. Two different microfluidic devices emulating the fast-flow and slow-flow compartments as well as IES of the spleen have been previously constructed and showed to retain parasitized, aged or defective red blood cells from hereditary spherocytosis^6,7^. Neither studies, however, reported observations on pitting casting doubts on whether this phenomenon is only due to the mechanical forces in the squeezing of infected-RBC through the slits.

In this study, we have designed and constructed two microfluidic devices with the objective of replicating the mechanical pitting functions of the spleen, which remove malaria parasites from infected RBCs, using a flow cytometry approach and imaging to evidence this process. We demonstrated that the microfluidic devices replicate ex vivo the pitting of *P.f.*-RBCs of the spleen by means of mechanical forces exclusively, leaving a population of once infected red blood cells, free from the parasite without causing cell rupture.

## Methods

### Sample preparation

Uninfected red blood cells (uRBCs) were obtained from donors at the Blood and Tissue Bank (Barcelona), with a written informed consent and in accordance with the Ethics Committee protocols of the Blood and Tissue Bank. Upon receipt, RBCs were washed twice with incomplete RPMI and resuspended to 50% hematocrit in the same medium. *P. falciparum*-infected RBCs (*P.f.*-RBCs) were obtained by culturing *P. falciparum* 3D7 parasites with human erythrocytes at 3% hematocrit in RPMI medium (Gibco 041-91762A) supplemented with L-Glutamine 200 mM (Invitrogen 25030-024). In all experiments, parasites obtained from in vitro cultures were concentrated using a 70% percoll gradient, followed by a 5% sorbitol treatment to differentially induce osmotic lysis of trophozoites and schizonts in order to achieve > 80% synchronous ring-stages^8^. Right before the experiment, RBCs were washed twice with incomplete RPMI and resuspended to 10% hematocrit in the same medium. Infections were characterized by optical microscopy of thin blood smears stained with Giemsa^8^ (see Supplementary Fig. S1). The experiments were conducted according to the guidelines of the Declaration of Helsinki and approved by the University of Barcelona Bioethics Commission (CBUB) (Project 160016 and date of approval March 1st 2016).

### Microfluidics, device design and fabrication

**Figure 1:**
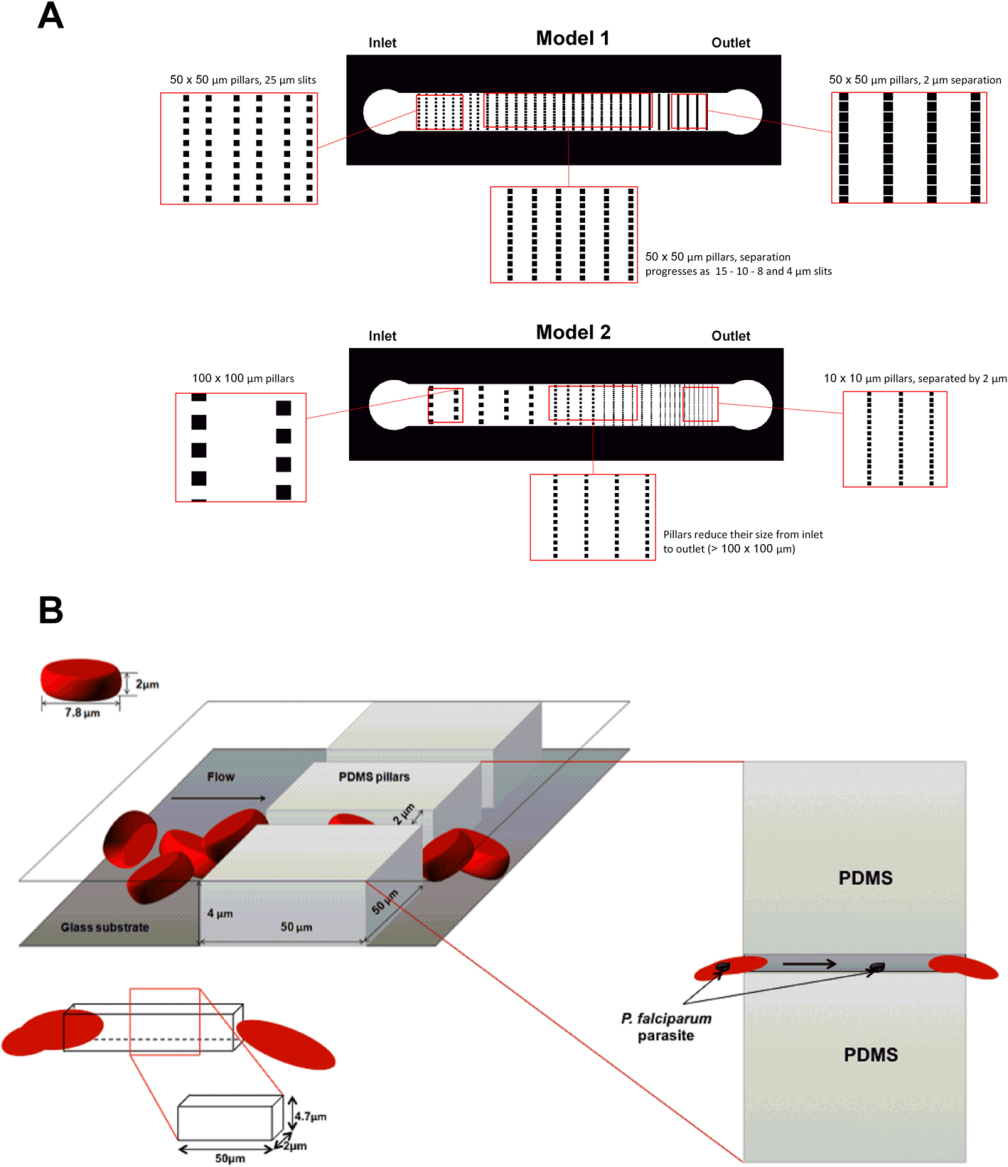
Microfluidic devices design for pitting studies. (**A**) Top: Device model 1. Single channel of 1 mm width × 8 mm length, with a filtration grid design of squared columns of 50 × 50 µm distributed along the channel, leaving a narrower space between them in each row (slits), from the inlet towards the outlet. Slits are 25, 15, 10, 8, 4, and 2 µm-wide, with a total of 100 slits progressively. Bottom: Device model 2. Single channel of 1 mm width × 8 mm length, with a filtration grid design of squared columns decreasing in size from 100 × 100 µm to 10 × 10 µm progressively, with a total of 498 slits. The space between columns in each row (slits) become narrower from the inlet towards the outlet, being the minimum slit size 2 µm-wide. (**B**) 3D representation of RBCs passing through 2 µm slits in the microfluidic channel. Inset shows the top view of the pitting process in which an infected RBC loses an intracellular parasite. *P.f.-*RBCs are depicted passing through a narrow path of 2 × 4.7 × 50 μm^3^. The design of the devices was made with Draftsight Software 2017 (Dassaults Systèmes) and the illustrations were created with power point.

**Figure 2:**
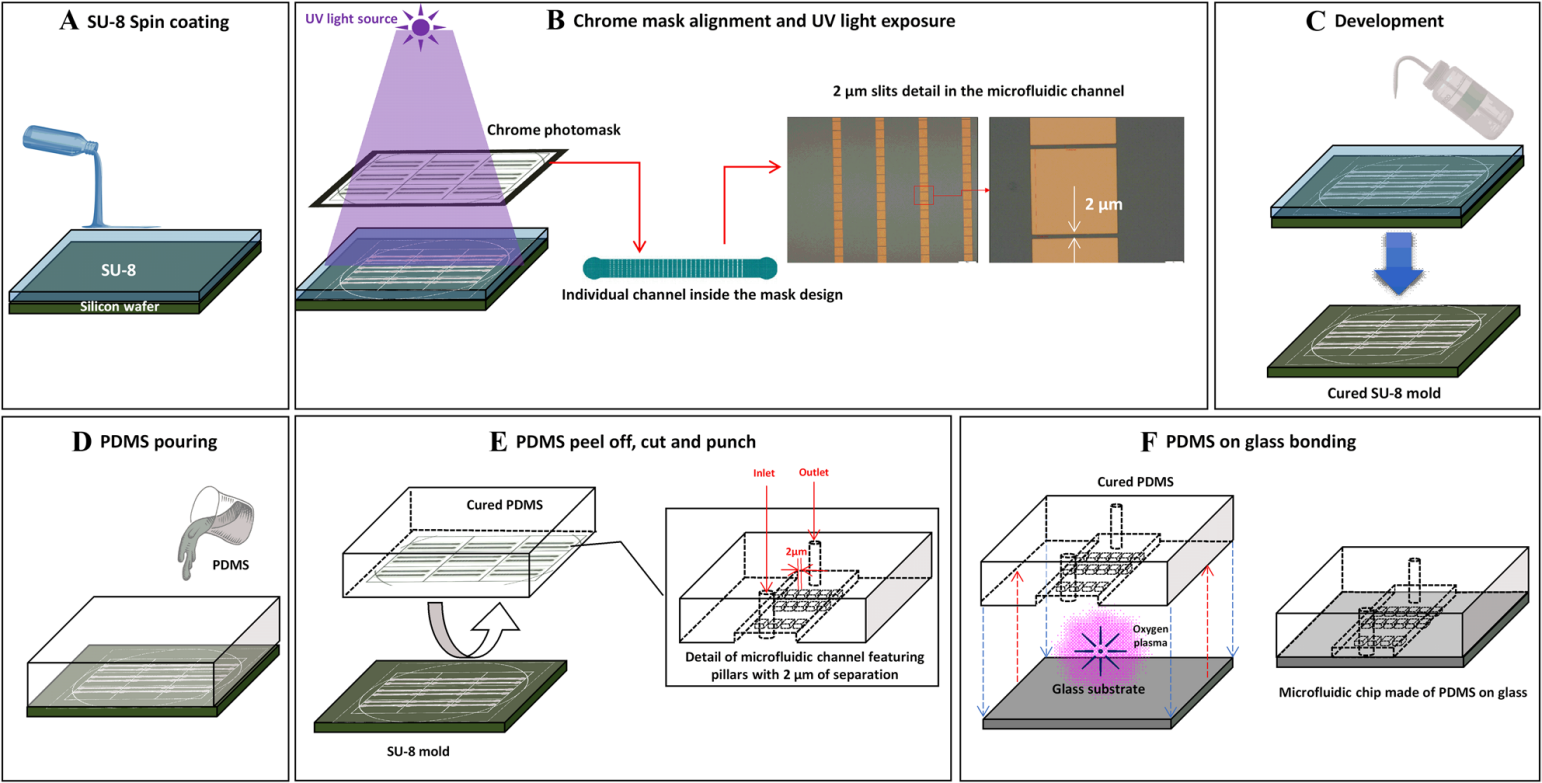
Workflow of microfluidic devices fabrication. (**A**) SU-8 Photoresin is poured over a silicon wafer. The material is evenly distributed on the substrate by spin coating. (**B**) A chrome photomask with the desired pattern is placed over the newly coated material. SU-8 is a negative photoresin, it polymerizes in presence of UV light. Sufficient exposure cures non covered areas by the mask. The cured sections resemble the shapes printed on the chrome mask, with a resolution of 2 µm. (**C**) Developing consists in washing off the non-cured SU-8 from the silicon wafer. This is done with developers specifically made for the photoresin in use. (**D**) PDMS mixed with curing agent at 10:1 proportion is poured onto the new mold. It is left to rest for at least 24 h or baked in the oven at 65 °C for 2 h. (**E**) The created structure is the negative version of the mold; this is the microchannel. After peeling it off, the PDMS channel is cut, inlet and outlets are punched. The PDMS channel is washed and dried thoroughly. (**F**) A clean glass substrate and the cured PDMS channel surfaces are exposed to oxygen plasma inside a plasma chamber. This changes the radicals on the surfaces, making it possible to bond both structures once they are put in contact facing with each other. The microfluidic channel is formed when the PDMS and the glass bond successfully. The illustrations were created with power point.

Two different models based on the principle of increasing the numbers of IES to achieve statistical significance of the passage of RBC through these slits as reported by Buffet and collaborators^7^ were designed and constructed (Fig. 1A). In both models, the grid of columns and slits were set on a rectangular microchannel of 1 mm width and 8 mm length. The slits width decreased uniformly along the microchannel from 100 µm to 2 µm. The difference between both designs is the dimension of their columns. Model 1 is composed of fix columns with dimensions of 50 µm × 50 µm and variations on the slit size from 50 to 2 µm, with a total of 120 slits. The large dimensions of the columns allow for the increase of time that cells need to pass through the slit, submitting the infected cell to a longer exposure to the deformability stress for pitting to take place. Model 2 is composed of different columns dimensions decreasing from 100 µm × 100 µm to 10 µm × 10 µm and slits width that decrease from 100 to 2 µm. This small column size has the advantage of increasing the number of constrictions per chip, 498 in total, therefore its performance relays on the high frequency of pitting. The design of the microchannels was developed using Draftsight Software 2017 (Dassaults Systèmes). The designs were printed, with high resolution, on a 10 cm diameter chrome glass mask. The microchannel fabrication was performed at the Physics Faculty of the University of Barcelona, using photolithography and replica molding techniques^9,10^ (Fig. 2). The masters were manufactured spin coating SU-8 negative photoresist (from Microchem) over a silicon substrate. We used 2 different photoresists SU-8 2010, to reach a microchannel depth of b = 7 µm, and SU-8 3005 to reach a microchannel depth of b = 4 µm. Silicon wafers were used instead of regular glass substrates, to ensure good adhesion of the photoresist. After the wafer was thoroughly covered with the photoresist, both mask and wafer were mounted on a lithography aligner. A controlled UV light source was activated, causing the polymerization process of the negative photoresist to take place. Being SU-8 a negative photoresist, the paths covered by the mask, which are not impacted by the UV light, were washed away in the development step. Once curing and baking recommended times were finished, the wafer was washed using the SU-8 developer. Finally, the entire uncured polymer was stripped away, leaving only the designs printed by the mask (see Supplementary Fig. S2A). A silanization process was carried out over the silicon surface after the photolithography process, in order to create a monolayer which protects the surface, prevents SU-8 structures from detaching and allows using the master several times without breaking the structures. The obtained wafers were covered with PDMS and set to solidify at 65 °C for 2 h. Once the PDMS was cured, it was carefully peeled off the wafer. Six sets of channels (three of each design) were cut using a sharp blade. The inlet and outlet for tubing were made using a punch with a diameter of 0.75 mm^2^. Newly cut and pierced PDMS channels were cleaned using water and ethanol to remove any grease or dust particles attached during handling. Next, they were rinsed using isopropanol and deionized water. Subsequently channels were blown dry using compressed nitrogen (see Supplementary Fig. S2B). Lastly, PDMS channels were bonded to clean glass substrates, using oxygen plasma, obtaining the microdevices (see Supplementary Fig. S2C).

### Experimental setup

**Figure 3:**
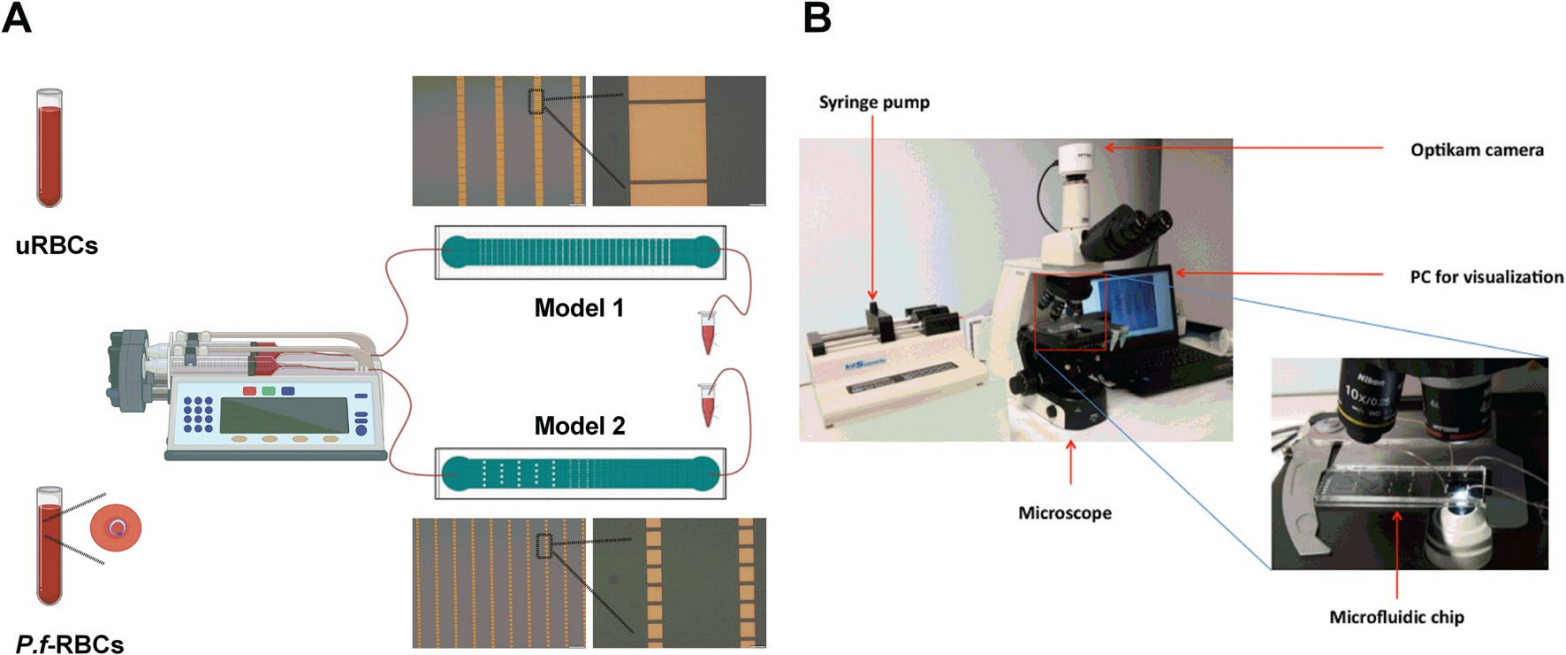
Pitting experimental setting. (**A**) Representation of uRBCs and *P.f.-*RBCs samples injection into the microfluidic devices and post-chip sample collection. (**B**) Images showing the equipment used for sample application into the devices connected to an optical microscope and the computer for visualization and recording. The drawings were created with Biorender.com.

A system consisting of 1 ml syringe (BD Plastipak) full of blood was connected through a needle (BD 25G 5/8′′, 0.5 × 16 mm) with Tygon tubing (Cole-Parmer, Illinois, USA) to the microfluidic device (input) The output of the microchannel was connected via a Tygon tube to a container in order to collect the blood that passed through the microdevice. The syringe was actuated with a KDS 200 series pump (kdScientific, catalogue no. 78-9202) (Fig. 3A,B). A constant flow of 5 μl/min was established in the microfluidic device. In all cases, blood passed just once through the devices. The experiments were carried out at RT (room temperature). An Optikam camera connected to an inverted microscope was used to record images (Fig. 3B).

### Hemolysis analysis

Hemolysis was quantified as a function of hemoglobin release in the medium supernatant after centrifugation of non-infected and *P.f.*-RBCs at 1000×*g* as previously reported^11^. For that, four conditions were tested for both infected and non-infected blood: (1) Fresh blood (negative control). (2) Blood passing through the devices at the physiological flow rate (5 μl/min). (3) Blood passing at 40 times the physiological flow rate (200 μl/min). (4) Cells permeabilized with saponin (0.15%) as positive control. Absorbance in supernatants at 540 nm was measured in a spectrophotometer (Thermo, Varioskan Flash).

### Pitting quantification by Flow cytometry

Cells from all experimental conditions were analyzed by flow cytometry to determine the proportion of pitted cells. Conditions included: (1) uRBCs and *P.f.-*RBCs not passing through any device, (2) uRBCs passing through both microdevice models and (3) *P.f.-*RBCs passing through both microdevice models. Three device replicates were used for both models and for both uRBCs and *P.f*.-RBCs in each experiment. After, cells were fixed with 4% paraformaldehyde − 0.075% glutaraldehyde in PBS at 4 ℃ for 30 min, and permeabilized with triton X-100 0.1% PBS at RT for 15 min. Next, cells were blocked with PBS-3% BSA for 1 h and, later incubated with mouse anti-RESA antibody (gently donated by Professor Klavs Berzins, Stockholm University, Sweden) (dilution 1:200) for 1 h at RT. After three washes in PBS, cells were incubated with the commercial secondary anti-mouse IgG conjugated with AlexaFluor 488 (Invitrogen A11008) (dilution 1:200) and DAPI (dilution 1:1000) at RT for 1 h. After cells were washed three times in PBS, samples were acquired on a BD FACSCanto™ II Flow Cytometer and analyzed with FlowJo software.

### Epifluorescence microscopy

Following the same staining protocol described above, RBCs were imaged in an epifluorescence microscope (Leica AF6000) before and after passing through the microfluidic devices.

### Image acquisition

Several movies 10 s-long of infected blood circulating through the slits were obtained using a laser scanning confocal microscope (TCS-SP5; Leica Microsystems) at a magnification of 25.0 × (water objective). Videos were analyzed using the ImageJ software (version 1.41n, Wayne Rasband, NIH).

### Statistical analysis

Data were analyzed using Microsoft Excel and GraphPrism statistics program. Hemolysis analysis were quantified using a Kruskal–Wallis test, considering statistical significance *p* value < 0.05. Flow cytometry pitting analysis were quantified using a *T* test with Wilcoxon signed-rank post-test, considering statistical significance a *p* value < 0.05.

## Results

### Microfluidic devices

We designed two microfluidic device models containing a set of pillars positioned at particular distance to replicate the minimum IES size that would force *P.f.-RBCs* to pass through the slits without collapsing, thus, simulating the pitting function of spleen (Fig. 1A). This could be achieved considering the cross-section of the slit to be smaller than the RBC surface. To avoid rotation of the cell, the height of the microchannels should not exceed the diameter of the cells. Hence, considering the average size of the red blood cells (7.8 µm diameter and 2 µm height), we defined two heights for the microchannels. The minimum slit cross-sections were approximately 5.75 and 3.41 times smaller than the area of the cell (Fig. 1B). The microfluidics devices were composed of microchannels with a filtration mesh structure, containing vertical square columns and slits that decrease in size along the microchannel and in the direction of the flow. The columns aim to obstruct the flow of the cells in order to decrease the number of RBCs, and observe them at the minimum slit zone at a smaller concentration. The minimum slit-wide zone of the design is where the microchannel slits will emulate the IES of the spleen, responsible for its filtration function.

The relationship between the path geometry and the flow from the system explains how the forces interacting in the fluid flow allow the pitting phenomenon to take place. The pressure drop $\Delta P$, inside the system can be expressed as the product of the fluid flow $Q,$ and the resistance $R$ posed by the microfluidic channel:

1 $$\Delta P = QR$$

where the flow rate $Q$ is defined as the product between the mean velocity of the fluid and the area of the cross-section of the channel, $Q = vA$. The area $A = \omega b,$ is a rectangular cross-section of width ($\omega )$ and height $\left( b \right).$ The resistance R is:

2 $$R = \frac{12\eta L}{{Ab^{2} }}$$

where $L$ is the lenth of the slit and $\eta$ is the viscosity of the fluid. Taking into account the equations stated above and knowing that the experimental set-up uses constant flow, a characteristic time is necessary to achieve pitting. This time depends on the velocity $v$. At the same time, it is dependent on the length $L,$ expressed in its relationship to parameter $R$. Choosing adequate dimensions for the microchannel, the selected length of the slits should be extensive enough to allow pitting to happen while avoiding hemolysis.

### Hemolysis

**Figure 4:**
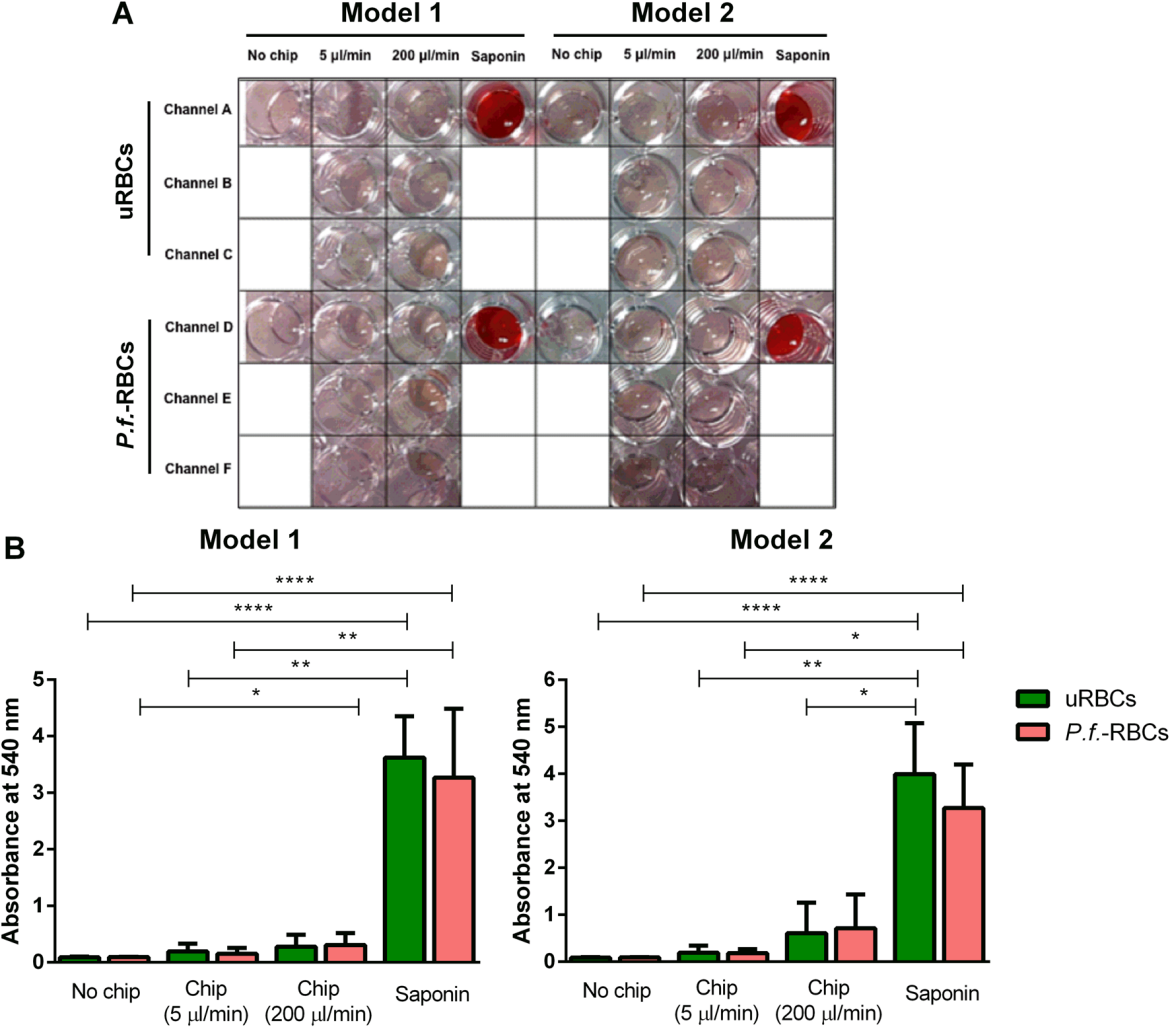
Hemolysis quantification of RBCs after passage through the microdevices. (**A**) Different conditions of the hemolysis assay with a picture of the 96-well plate containing the supernatants of the different samples. Data correspond to one representative experiment. (**B**) Hemolysis quantification of RBCs. Plots show the mean and the standard error of supernatant absorbance values of uRBCs and *P.f.-*RBCs that passed through model 1 and model 2. Data correspond to all experiments performed through the study. Statistical significance was determined using the Kruskal–Wallis test. Dunn's multiple comparisons test showed the following results (****p ≤ 0.0001; ***p ≤ 0.001; **p ≤ 0.01; *p ≤ 0.05).

Hemolysis experiments were conducted to exclude the possibility that the flux through the microchannels caused the rupture of RBCs during its transit through the slits. For that, spectrophotometric quantification of released hemoglobin in the cells medium was performed^11^. Visual inspection (Fig. 4A) and statistical analysis of hemolysis quantification by spectrophotometry (Fig. 4B) showed that non-infected and *P.f.-*RBCs after passing through both device models were intact as a significant difference in the hemoglobin release was found when compared with saponin-treated RBCs, used as a positive control of hemolysis. Importantly, we did not detect hemolysis of RBCs passing throughout the devices at physiological flow rate (5 µl/min), neither at 40 times the physiological flow rate. These data indicate that RBCs remain intact after passing through the slits of both microfluidic devices designed.

### Pitting quantification by flow cytometry

**Figure 5:**
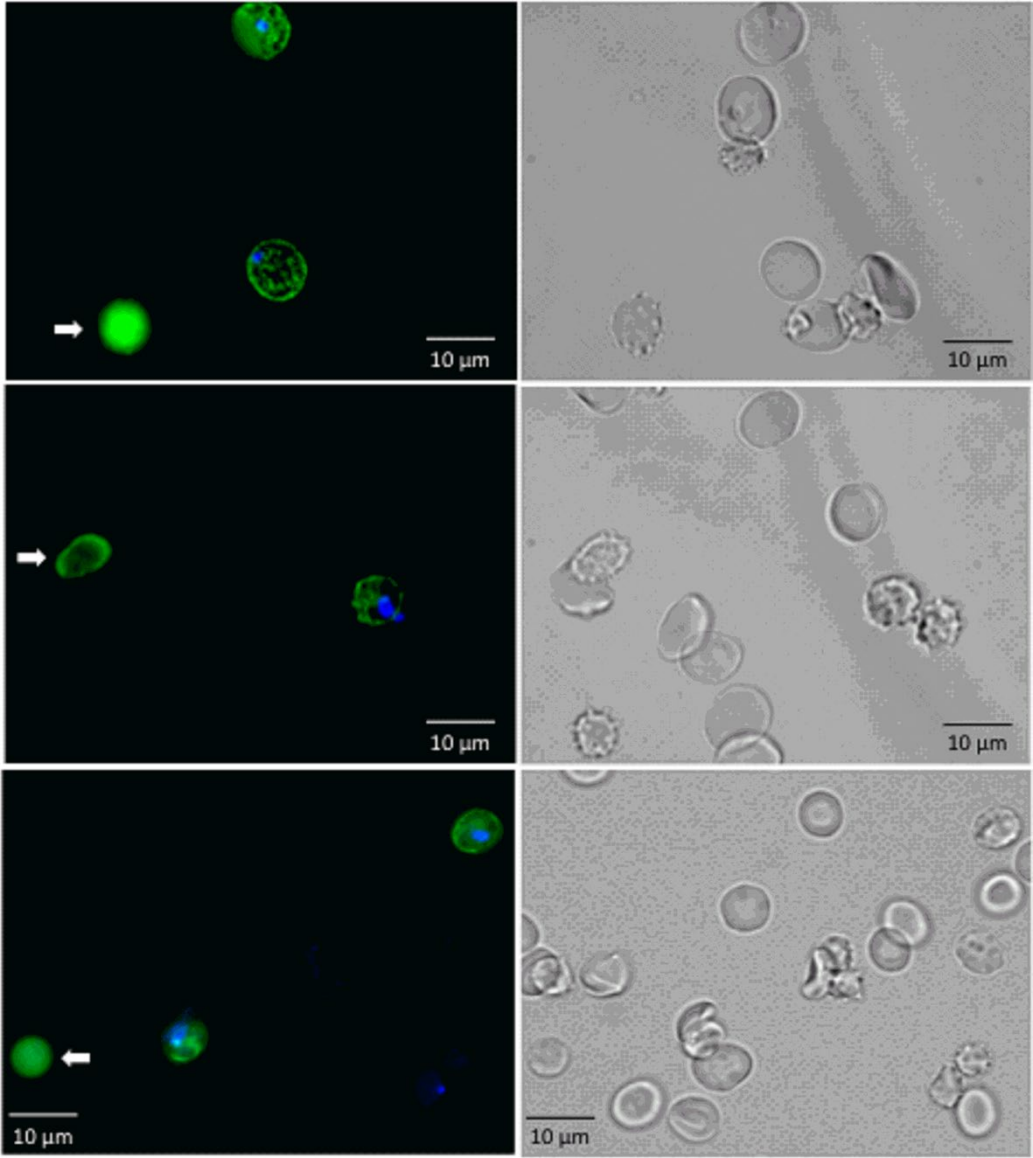
Fluorescence microscopy images of *P.f.-*RBCs after passing through the microfluidic devices (left column). Infected cells are shown as DAPI^+^ RESA^+^ (blue and green). White arrows indicate pitted cells (DAPI^-^RESA^+^, green). Corresponding bright field images of the same fields are shown in the right column. The images were obtained with an epifluorescence microscope (Leica AF6000) and modified using ImageJ software (version 1.41n Wayne Rasband, NIH, (https://imagej.nih.gov/ij/).

We employed the microfluidic devices to study the pitting of *P.f.-*RBCs using an approach based on flow cytometry. *P.f.-*RBCs containing intact parasites are expected to be double-stained with RESA antibody, which binds to RESA protein exposed in the infected RBC surface, and with DAPI, which stains parasite’s DNA. By contrast, pitted infected RBCs (O-iRBCs), are expected to lose parasite DNA staining while keeping RESA on their surface. In order to confirm that our staining approach for distinguishing *P.f.-*RBCs with RESA antibody and DAPI was suitable for this purpose, we first confirmed the staining pattern of these markers by fluorescence microscopy in infected cells after passing through microfluidic devices. We observed several cells showing a double-stain pattern of RESA and DAPI labelling parasite’s surface and DNA, respectively, in *P.f.-*RBCs. A small proportion of cells with single staining of RESA was also observed, indicating the presence of O-iRBCs and confirming therefore the expected staining pattern (Fig. 5).

Next, we performed a pitting quantification analysis by flow cytometry using a gating strategy to define uRBCs (DAPI^-^RESA^-^), *P.f.-*RBCs (DAPI^+^RESA^+^) and O-iRBCs (DAPI^-^RESA^+^) (see Supplementary Fig. S3A). Importantly, we observed that in all samples, and more prominently those that passed through the devices, independent of its infection status, a population of DAPI^-^RESA^medium^ was detected, accounting for approximately 0.5% of total cells. Based on the high fluorescence intensity of RESA marker that we observed in *P.f.-*RBCs population after passing through the device, which clearly differentiated from this DAPI^-^RESA^medium^ observed in uRBCs, we redefined the truly O-iRBCs population as DAPI^-^RESA^high^, therefore excluding this background population from the pitting analysis. Using this gating strategy, we quantified the basal frequencies of all above-mentioned populations in infected cultures from seven independent experiments before passing through the devices. Results showed a great reproducibility in the proportion of all populations (see Supplementary Fig. S2B), indicating that infections in the different experiments where homogeneous with 10% of total cells accounting for *P. falciparum* infected RBCs.

**Figure 6:**
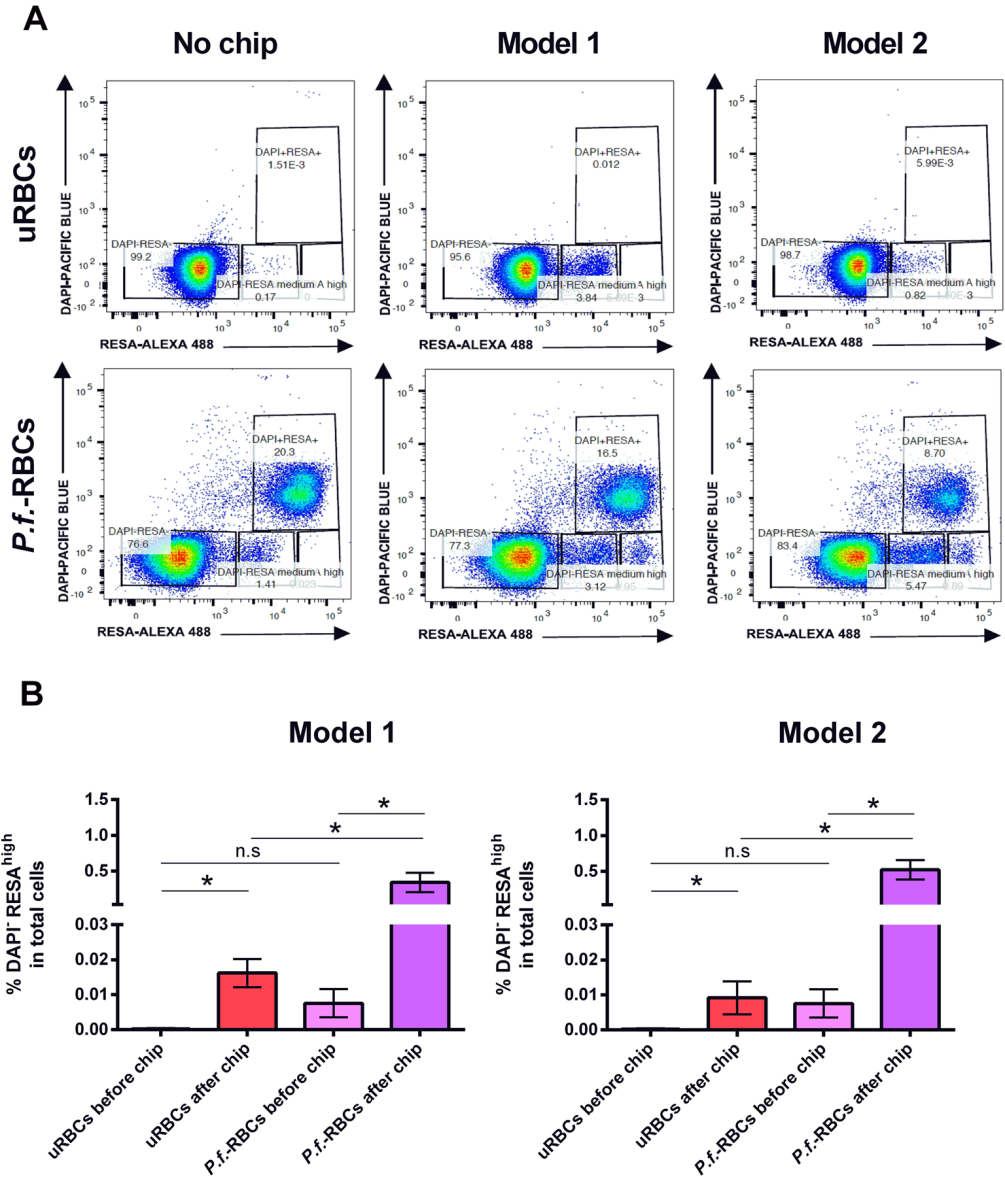
Flow cytometry-based pitting quantification. uRBCs and *P.f.-*RBCs were collected and analyzed by flow cytometry before and after passing through the microfluidic devices. Cells were gated according to the strategy described in Supplementary Fig. S3. (**A**) Representative dot plots of uRBCs (DAPI^-^RESA^-^), autofluorescent uRBC (DAPI^-^RESA^medium^), *P.f.-*RBCs (DAPI^+^RESA^+^), O-iRBCs (DAPI^-^RESA^high^) before (No chip) and after passing through microfluidic device models 1 and 2. This figure corresponds to one experiment out of seven. Twelve devices were used in each experiment (six of model 1 and six of model 2). (**B**) Frequencies of DAPI^-^RESA^high^ cells from model 1 and model 2 between uninfected and infected cells before and after passing through the devices were compared. Data represent the mean and standard error of the mean obtained from seven independent experiments. Statistical significance was assessed by a *t* test with Wilcoxon signed-rank post-test, being considered statistical significance (*p value < 0.05).

Pitting analysis consisted in comparing DAPI^-^RESA^high^ population in *P.f.*-RBCs before and after passing through the device. We also included in this comparison uRBCs before and after passing through the device to exclude any possible confounding product of alterations in RBCs due to the mechanical passage through its slits (Fig. 6A). Beside the very low frequency of DAPI^-^RESA^high^ population in all samples, results from seven independent infections showed that frequencies of pitted uRBCs and O-iRBCs (DAPI^-^RESA^high^) after passing through both device models were significantly higher when compared to the proportion of this cell population before its passage through the microchannels (Fig. 6B). This was done for the twelve devices used in each experiment. The finding that uRBCs, which by definition are DAPI^-^RESA^-^, become DAPI^-^RESA^high^ upon its passage through the device was unexpected and probably indicates that some cellular alterations are induced by the mechanical passage of the cells that makes them autofluorescent or prompt to unspecific binding of RESA antibody to its membrane. Nevertheless, when we compared frequencies of “pitted” uRBCs (DAPI^-^RESA^high^) with truly *P.f*.-RBCs (DAPI^-^RESA^high^) we found a significant higher (*p* < 0.05) (22 fold) increase of pitted *P.f*.-RBCs after passing through both device models (Fig. 6B). These results suggest that the passage of *P. falciparum*-infected RBCs through the microdevices slits caused the loss of parasites, leaving once-infected RBCs expressing RESA antigen on their surface, thus, evidencing the mechanical pitting phenomenon of malaria infected RBCs and supporting our theoretical prediction of suitability of these devices for modelling the pitting function of the spleen.

### Imaging

**Figure 7:**
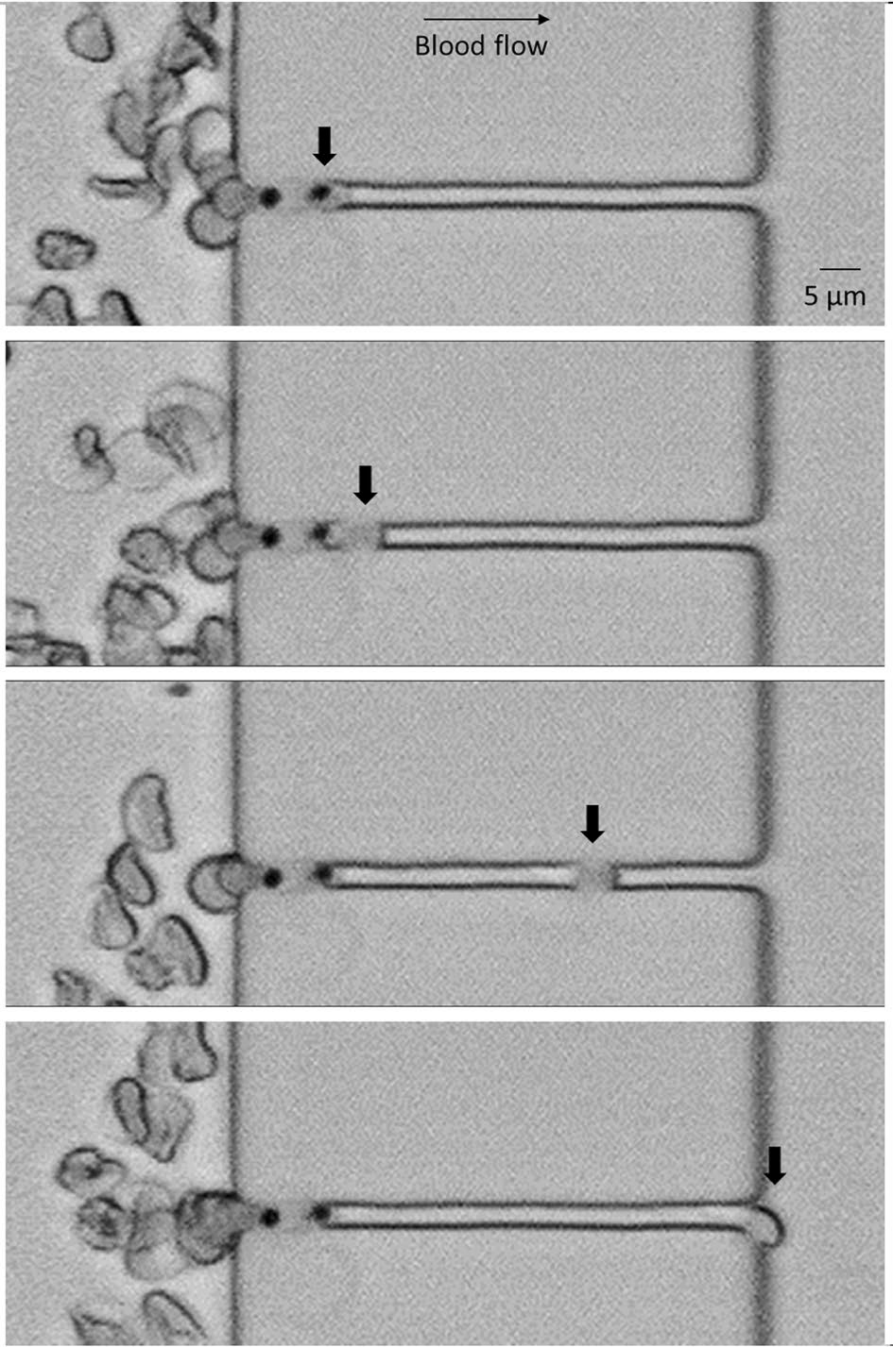
Time lapse-series showing an iRBC undergoing the pitting process during the passage through a 2 µm slit of a model number 1 device. In the upper picture, a *P.f.-*RBC (thick arrow) is embedded into the slit. In the subsequent below, the parasite separates from the rest of the cell, which continues flowing easily through the slit. A laser pictures scanning confocal microscope (TCS-SP5; Leica Microsystems) at a magnification of 25.0 × (water objective) was used to record movies from which images were extracted. The pictures were modified using ImageJ software (version 1.41n Wayne Rasband, NIH, (https://imagej.nih.gov/ij/).

In order to visualize the pitting process occurring in *P.f.-*RBCs flowing through the slits of the devices, we recorded several movies in the laser scanning confocal microscope. The compilation of movies recorded showed systematically RBC deforming while passing through the smaller slits (2 µm-wide) (Fig. 7 and Supplementary Movie 1). Of note, *P.f.-RBCs* were distinguished by the presence of hemozoin, seen as an intracellular dark dot in the infected cells. Remarkably, we observed in a number of cells the retention of such dots in the slits and the exit of empty O-iRBC from these slits during its passage through the devices (Fig. 7), consistent with the results obtained in the pitting quantification by flow cytometry (Fig. 6). These results strongly suggest that pitting of *P. falciparum-*infected RBCs ring stages through IES of the spleen can be achieved by mechanical constraints not needing phagocytic cells.

## Discussion

Here we constructed two different devices to specifically study the pitting function of the spleen where a single fluid flow, only emulating the slow-flow compartment of the spleen, forces RBCs to sequentially pass through slits of decreasing sizes. Pitting is defined as the removal of solid particles from the cytoplasm of a red cell without destructing the cell. Originally observed in hereditary non-spherocytic hemolytic anemia^12^, the presence of other red cell particles such as red-cell nuclei, Heinz bodies and Howell-Jolly bodies, as well as malaria parasites and Bartonella in splenectomized patients^2^, indicates that pitting is a corrective action in red blood cell disorders, such as hemolytic anemias.

Two different microfluidic devices mimicking the minimal functional filtering unit of the spleen have been previously reported^6,7^. In the first one, emulations of the 90% fast-flow and 10% slow-flow human spleen compartments as well as IES, were used to demonstrate the filtering capacity of the spleen^6^. Aged and *P. yoelii*-infected reticulocytes passing through this device using physiological flow-rates indeed demonstrated that aged cells got significantly trapped in the IES as opposed to the more flexible infected-reticulocytes. A limitation of such device, however, was the low numbers of IES, thus limiting the statistical power to reach biological meaningful numbers of retention/passage of aged or infected cells. To further study spleen IES retention with statistical power, another device was designed and constructed containing eight filtering units each comprising more than fifty 2 µm slits^7^. Using such device, it was elegantly demonstrated that heat-treated RBCs, *P. falciparum*-infected RBC and cells from patients with hereditary spherocytosis were significantly retained, albeit variably, in their passage through the IES. Neither studies, however, reported observations on pitting casting doubts on whether this phenomenon is only due to the mechanical forces in the squeezing of infected-RBC through the slits.

Due to changes on the mechanical properties of the RBCs forced through the microfluidic devices, precisely the total number of 2 µm slits is 120 for model 1 and 498 for model 2 (Fig. 1), it was of upmost importance to demonstrate that hemolysis was not a confounding factor when studying pitting in these devices. Hemolysis analysis of uRBC and *P.f.-*RBCs, indeed showed that the passage of cells through the microchannels allows their deformation and constriction without causing rupture, validating thus the use of these devices for pitting studies (Fig. 4). However, the analysis of pitting by flow cytometry showed that a small fraction of uRBCs become fluorescent after its mechanical passage of cells through the channels, probably indicating some degree of cell damage that may have caused unspecific binding of antibodies to its cell membrane or death-induced autofluorescence, altering scatter properties and generating a once-infected red blood cell, “O-iRBC phenotype” (DAPI^-^RESA^high^). It is already known that autofluorescence commonly overlaps with the spectrum of research fluorophores, and consequently interferes with the fluorescent microscopy and cytometric analyses^13^. In spite of this unwanted effect, statistical analysis demonstrated that both devices modelled the mechanical removal of *P. falciparum* parasites, as was shown by flow cytometry analysis (Fig. 6). In addition, fluorescence (Fig. 5) and light microscopy (Fig. 7 and Supplementary Movie 1) also demonstrated that pitting of *P. falciparum* in the spleen can occur by mechanical forces.

The product of pitting is the creation of surface area depleted RBCs^2^ termed spherocytes, a term that indicates cells that are less disc like than normal RBCs. Very relevant, in patients with malaria the clearance of blood-stage parasites is faster after treatment with artemisinins than with other antimalarial drugs (e.g., quinine). Artemisinins act very early in the parasite’s asexual life cycle where young parasite forms (rings) are cleared before they mature to potentially pathogenic cytoadherent forms^14^. These two pharmacodynamics effects, which are not achieved by quinine, are believed to reduce the morbidity and mortality of severe malaria in Southeast Asia and sub-Saharan Africa^15,16^. Newton and collaborators observed that the O-iRBC count rise was faster for patients treated with an artemisinin derivative than those treated with quinine, which probably reflects the greater efficacy of artemisinin derivatives in killing or damaging circulating ring-stage parasites^17^. *P. falciparum* rings exposed to artemisinins appear morphologically similar to Howell–Jolly bodies and are expelled from their host RBC in the same way. Consistent with these similarities, pitting of infected RBCs does not occur in splenectomized patients or in in vitro culture. Ndour and collaborators analyzed the pitting rate of *P.f.*-RBC after exposing them to artesunate in vitro by filtering them through microsphere layers, which means that mechanical interactions are involved in pitting^18^.

It is worth of mentioning that the pitting rate concept, defined by Buffet and collaborators^19^ in the context of malaria is different from the classical rate of circulating pitted RBCs^2^. Actually, it is of importance to make a clear distinction between both terms because they imply opposite situations. The classical concept of pitting, or culling, is used as the gold standard for assessing the filtering function of the spleen in patients with potential asplenia^2^. It results from the removal of (non-microbial) intracellular remnants by macrophages as evidenced by small surface depressions that can be visualized using differential interference microscopy (Nomarsky optics). Because this process removes part of the RBC membrane, pitted RBCs become less deformable and are cleared from the circulation by the spleen. Thus, a rate of circulating culled RBCs too high is an indication of a higher risk for overwhelming infection due to functional asplenia^2^. By contrast, in the context of malaria patients, RBCs from which parasites have been removed (or pitted) are called once-infected red blood cells (O-iRBCs) and visualized as RESA positive cells lacking the parasite nucleus (DAPI negative)^19^. Since O-iRBCs are not detected in the peripheral blood of asplenic malaria patients, their presence is a marker of spleen-clearing functions.

Future functional studies of the collected DAPI^-^RESA^+^ cells (O-iRBCs) after passing through the device would help answering the question about function preservation of these cells. Theoretically and from the evolutionary point of view, the main goal of the pitting process is believed to mitigate the anemia caused by the RBC destruction in the infection process. This way, the O-iRBCs would continue to be functional, at least for some more time, and oxygen transportation would not be as affected as if these previously parasitized cells were destroyed^20^. Indeed, this extra time granted has been calculated and the mean lifespan of these “deparasitized” RBCs is 7.6 days, compared with a mean RBCs lifespan of 43 days^21–23^. Removal of the parasite from the erythrocyte and subsequent phagocytosis, seems to be a more-efficient defense mechanism than ingesting the infected erythrocyte. This mechanism of destroying the parasite is common, and it may be particularly important during high parasitemias when the prevention of erythrocyte destruction is beneficial to the host^23^. Moreover, because pitted erythrocytes are derived from cells parasitized with ring stages, which consume little hemoglobin, it is likely that these salvaged cells can contribute to the transport and delivery of oxygen and carbon dioxide, attenuating the consequences of parasitism^17^.

To overcome some of the limitations of this study, we contemplate as a future work increasing the number of experiments included, which will be better to find differences in the statistical analysis. Also, the use of freshly drawn blood from patients with malaria in different disease stages, which may have consequences concerning cell membrane flexibility. These issues could be addressed in the future by testing the devices in the field. Finally, we seek to perform experiments with blood samples from splenectomized patients and patients with other hemolytic anemias, so that the pitting performance will be analyzed in other cell inclusion particles.

## Conclusions

We have developed a microfluidic device that replicates ex vivo the mechanical pitting of *P. falciparum*-infected RBCs of the spleen leaving a population of once infected RBCs deparasitized. This demonstrates that pitting can be achieved without the involvement of phagocytic cells by means of mechanical forces. Although pitting is a classic concept in the literature, little is known about the details of this process itself. Thanks to this work, we have now a better understanding of this process, both from the physical and biological point of view. Furthermore, we have been able to replicate the pitting of *P.f.-* RBCs thanks to the portable microfluidic devices, which prompts us to think that our design thus has applications for exploring pathophysiology in malaria and other hemolytic anemias where pitting corrects for red blood cells with solid particles in the cytoplasm without destructing the cell.

## Supplementary Information

Supplementary Video 1.

Supplementary Video 2.

Supplementary Information 1.

## Data Availability

The data are available by contacting the corresponding authors.
