# Supplementary material for: Pitting of malaria parasites in microfluidic devices mimicking spleen interendothelial slits
Source: Sci Rep. 2021 Nov 11;11:22099. doi: 10.1038/s41598-021-01568-w (PMC8585870; doi:10.1038/s41598-021-01568-w)
Supplement: Supplementary file 3 — Supplementary Information 1. [file 41598_2021_1568_MOESM3_ESM.pdf]

SUPPLEMENTARY INFORMATION (Elizalde-Torrent, Trejo-Soto et al.)

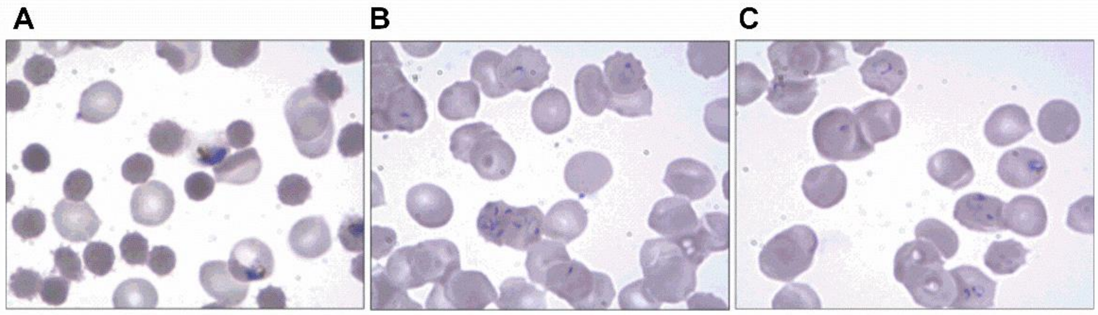

Figure S1

Figure S1. Light microscopy images of Giemsa stained *P.f.*-RBCs.

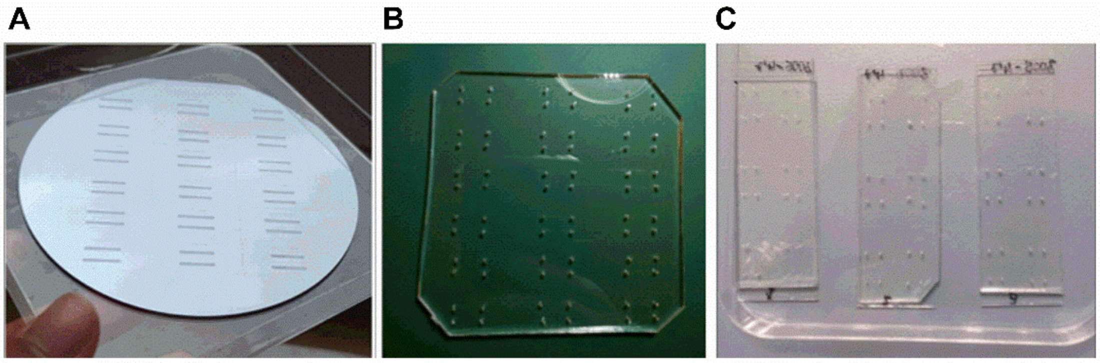

Figure S2

Figure S2. Microfluidic prototype devices images at different fabrication steps. (A) SU-8 mold on silicon wafer. (B) PDMS channel, after being peeled off the mold. (C) Microfluidic channels consisting of PDMS on glass.

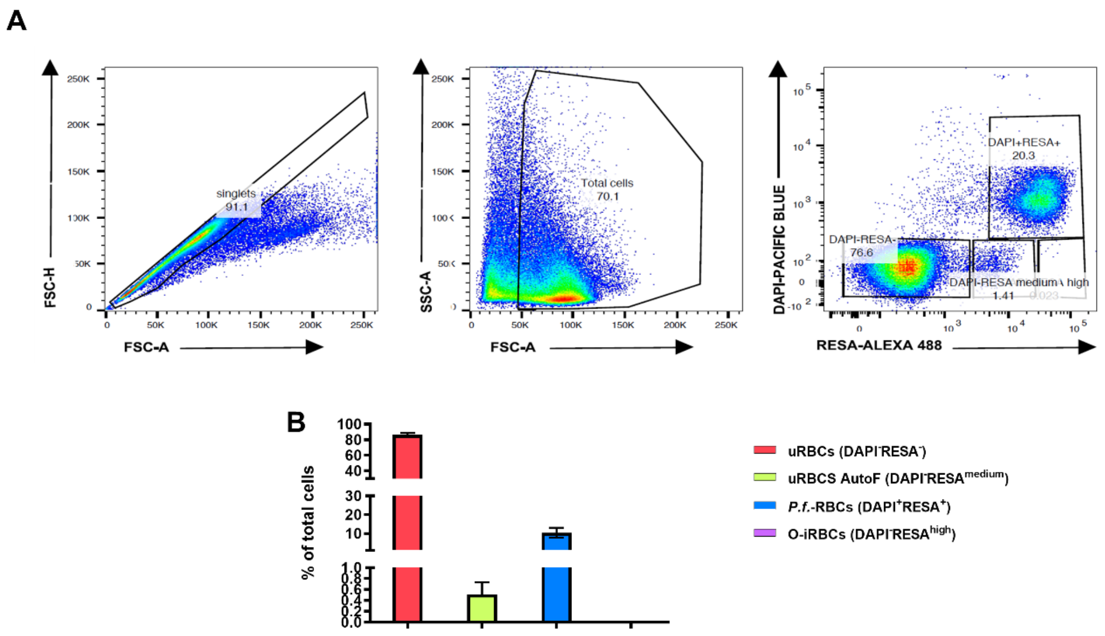

Figure S3

Figure S3. Pitting flow cytometry analysis. (A) Gating strategy used for flow cytometry analysis of *P.f.*-RBCs pitting. Singlets were selected by gating events in the diagonal of FSC-A *vs* FSC-H. Total cells were selected based on morphology in the FSC-A and SSC-A. Cell populations were defined as: uRBCs (DAPI$^-$RESA$^-$), autofluorescent uRBC (DAPI$^-$RESA^{medium}), *P.f.*-RBCs (DAPI$^+$RESA$^+$), O-iRBCs (DAPI$^-$RESA^{high}). (B) Quantification of frequencies of the above-mentioned populations in *P.f.*-RBCs before passing through the devices. Data represent the mean of seven independent experiments.

MOVIE 1. Pitting movie for device model 1.mp4. The video shows an iRBC undergoing the pitting process during the passage through a 2 µm slit of a model number 1 device (50 x 50 µm columns). The movie was obtained with a laser scanning confocal microscope (TCS-SP5; Leica Microsystems) at a magnification of 25.0× (water objective). The video was edited with ScreenCast-O-Matic (version 2.9.2).

MOVIE 2. Pitting movie for device model 2.mp4. The video shows an iRBC undergoing the pitting process during the passage through a 2 µm slit of a model number 2 device (10 x 10 µm columns). The movie was obtained with a laser scanning confocal microscope (TCS-SP5; Leica Microsystems) at a magnification of 25.0× (water objective). The video was edited with ScreenCast-O-Matic (version 2.9.2).
